# Bimetallic Gold Nanostars Having High Aspect Ratio Spikes for Sensitive Surface-Enhanced Raman Scattering Sensing

**DOI:** 10.1021/acsanm.2c02234

**Published:** 2022-08-29

**Authors:** Supriya Atta, Tuan Vo-Dinh

**Affiliations:** † Fitzpatrick Institute for Photonics, Duke University, Durham, North Carolina 27708, United States; ‡ Department of Biomedical Engineering, Duke University, Durham, North Carolina 27708, United States; § Department of Chemistry, Duke University, Durham, North Carolina 27708, United States

**Keywords:** gold nanostars, silver coated gold nanostars, bimetallic, high aspect-ratio spike, SERS, sensing

## Abstract

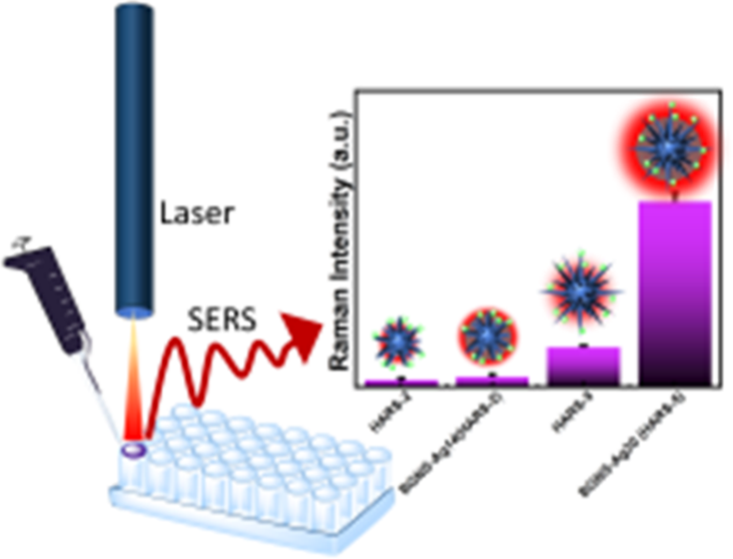

There has been increasing interest in evolution of plasmonic nanoplatforms based on noble metal nanoparticles to achieve ultrasensitive detection of trace analyte molecules through solution-based surface-enhanced Raman spectroscopy (SERS). This work presents a surfactant-free synthesis method of bimetallic gold nanostars coated with silver (BGNS-Ag) having sharp, high aspect-ratio spikes for achieving ultrahigh detection sensitivity and high reproducibility. Specifically, the unique BGNS-Ag platform combines both the strong SERS enhancement effects of gold nanostar sharp spikes and the high scattering feature of the silver–gold bimetallic structure. To achieve SERS reproducibility, this solution-based SERS measurement requires minimal sample preparation without addition of any external reagents, which can cause irregular aggregation of nanoparticles and reduce the reproducibility of SERS measurements. Moreover, we have streamlined our SERS sensing procedure by using standard well-plates and a portable Raman device for SERS measurements, which could be utilized for rapid on-site detection. This solution-based SERS performance was studied using methylene blue (MB) as a model analyte system. The detection limit of MB was as low as 42 pM, indicating high sensitivity of detection using BGNS-Ag. To illustrate the usefulness for environmental sensing, we showed that the SERS sensor can detect a pesticide, thiram, at a concentration as low as 0.8 nM. This study demonstrated that the BGNS-Ag system could serve as an effective and versatile plasmonic-active platform for reproducible, fast, and in-field detection of small organic analytes at trace levels.

## Introduction

Raman spectroscopy has received considerable interest for the identification of various types of analyte molecules, including illicit drugs, pesticides, food contaminants, and so forth, due to its unique vibrational patterns reflecting their molecular structure, named “molecular fingerprint.”^1−4^ However, the sensitivity of Raman spectroscopy suffers from a very poor detection limit due to the low scattering cross-section of Raman active molecules.^1^ SERS is produced by enhancement of the Raman signal of a Raman active molecule that is close to the surface of a plasmonic metallic nanostructure. Fleishmann and co-workers first reported this phenomenon in 1974.^5^ This enhancement was further confirmed by Jeanmaire and Van Duyne^6^ and by Albrecht and Creighton.^7^ Subsequently, our lab first introduced SERS as an analytical tool in 1984.^8^ For over three decades, we have developed different SERS platforms for many applications in environmental monitoring, biological sensing, medical diagnostics, and therapy.^9−15^ To date, the solid substrate-based SERS detection method has been well established and commercialized.^16,17^ However, this method has many shortcomings, such as the need for skilled personnel, elaborate sample processing, and time consuming procedures. Therefore, an easy, rapid, and sensitive SERS detection platform with good reproducibility and minimal sample preparation is essential, which would lead to many important real-world applications.

The solution-based SERS detection technique could offer great prospects for easy and rapid detection.^18^ Unfortunately, solution-based SERS has achieved little success as an analytical technique because of relatively poor sensitivity, which is probably due to the dynamic and competitive interaction of analyte and solvent molecules with the SERS substrate.^19^ For the past few years, extensive efforts have been devoted improving the solution-based SERS performance, including surface modification and introducing external reagents.^20−25^ Among these, a commonly used approach involves the introduction of salts, which are known to induce aggregation of nanoparticles and enhance SERS signals.^26^ However, these methods are not suitable for practical applications because of uncontrollable aggregation resulting in low reproducibility of SERS signals.^21,27^ Therefore, it is highly desirable to develop a hybrid nanomaterial system which can exhibit high sensitivity without any sample pretreatment.

Anisotropic gold nanostars (GNS) have attracted great interest because of their sharp protruding spikes, which exhibit strong enhanced electromagnetic fields induced by localized surface plasmon resonance (LSPR), known as “hot spots.”^28−31^ For these reasons we first introduced GNS as a platform for SERS detection.^32^ GNS shows a higher SERS enhancement in comparison to other shapes of gold nanoparticles such as gold nanospheres, nanoshells, and nanorods.^33^ There has been a great success in substrate-based SERS detection by utilizing GNS, where a femtomolar detection limit was achieved.^34^ Unfortunately, the solution-based SERS detection limit of GNS is very low.^20^ We have previously performed finite element modeling of GNS and reported that the electric field is expected to be highest at the sharp tips of GNS because of large surface charge density, which indicates that it is important to tune the morphology of GNS with sharp multiple branches in order to achieve maximum SERS enhancement.^35^ Therefore, an improved synthesis approach for morphological modification of gold nanostars is required to enhance the SERS signal. Indeed, our group has first developed the surfactant-free GNS synthesis method and extensively explored the synthesis parameters to tune the GNS morphology for better SERS sensing.^29,36^ Recently, silver-coated small-sized GNS has been developed, which shows great potential as a SERS substrate where the high plasmonic effect of Ag has been utilized.^37,38^ Unfortunately, the GNS were stabilized by a surfactant cetyltrimethylammonium bromide (CTAB), which is necessary to protect the highly energetic sharp tips of the GNS, but it has a negative impact on SERS as it prevents analyte molecules from getting closer to the gold surface. Therefore, it is important to develop a new synthesis method to coat silver on surfactant-free GNS having sharp tips and densely branched morphology.

**Scheme 1:**
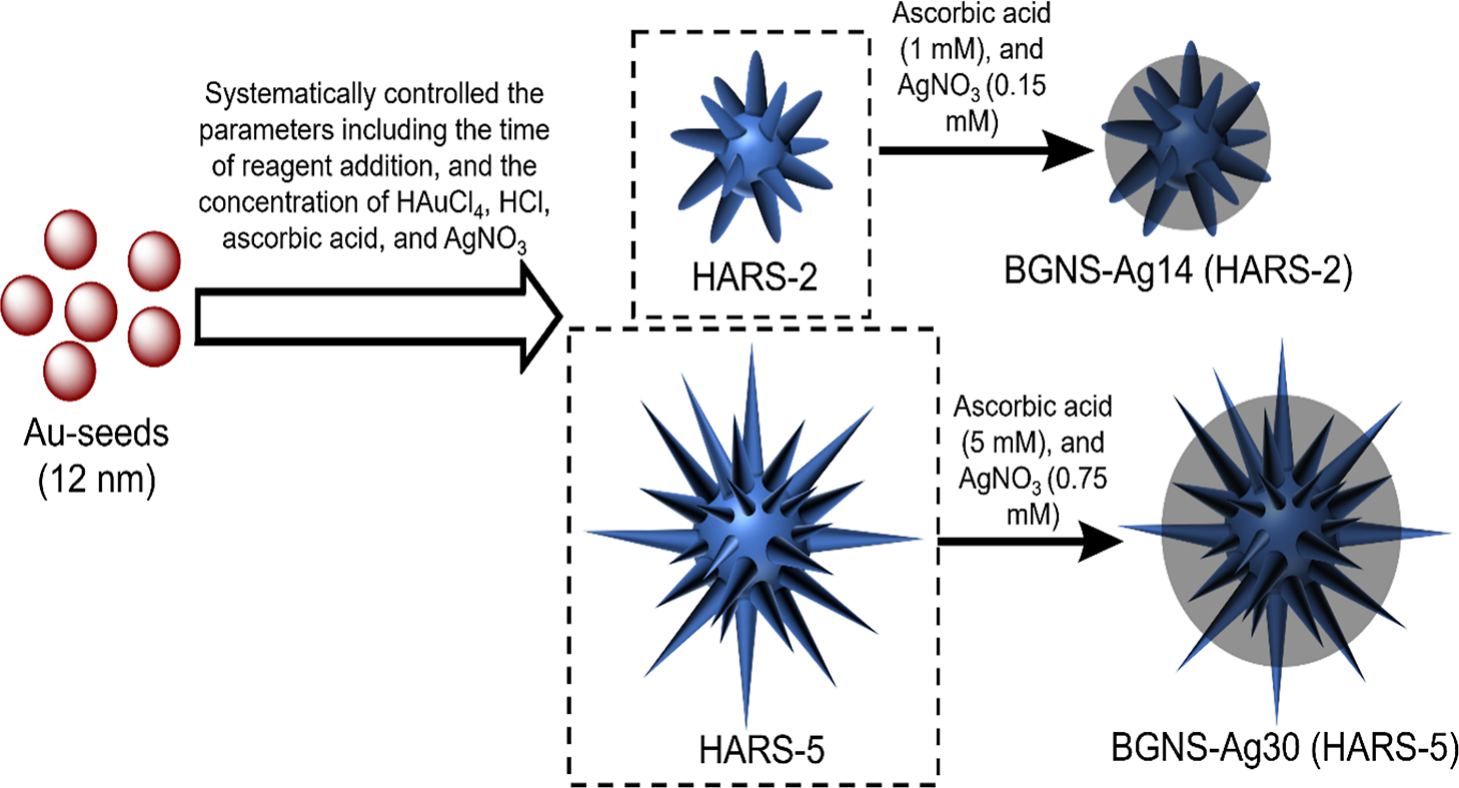
Schematic Representation of GNS Synthesis, Which Shows That the HARS Morphology Can be Tuned from HARS-2 to HARS-5 by Systematically Controlling the Time of Reagent Addition and the Concentration of HAuCl_4_, HCl, Ascorbic Acid, and AgNO_3_, and Ag Coating of HARS-2 and HARS-5 with Retained Morphology of Sharp Spikes of GNS by Employing Ascorbic Acid as the Reducing Agent

In the present work, we report a facile method for the synthesis of BGNS-Ag with high aspect-ratio spikes (HARS), as shown in Scheme 1. The high aspect-ratio gold nanostars were selected, in which many sharp spikes not only increase the surface area but also give rise to hot spots, which could enhance SERS signals. Subsequently, the reduced Ag atoms selectively deposited at the core of high aspect-ratio gold nanostars generate multiple hotspots, leading to an ultrahigh SERS signal. Moreover, by changing the amount of AgNO_3_, the plasmon resonance was facilely tuned. Moreover, we have utilized standard well-plates for SERS performance as a cost-effective approach.

## Results and Discussion

The main experimental concept for an efficient solution-based SERS substrate is presented in Scheme 1, which involves two steps. In the first step, surfactant-free GNS with densely populated HARS were synthesized, which would provide an intense electromagnetic field at the sharp tips and enhance the SERS signal. A surfactant-free GNS synthesis method was chosen instead of using polymers (e.g., poly(vinylpyrrolidone), PVP) or surfactants (e.g., cetyltrimethylammonium bromide/chloride, CTAB/CTAC) to achieve maximum SERS signal as SERS probe molecules can get a closer contact to the sharp spikes of surfactant-free GNS.^39^ Herein, two different branch aspect-ratios of GNS, referred to as HARS-2 and HARS-5 (Scheme 1) were synthesized. In the second step, we have investigated the silver coating of the HARS morphology by employing ascorbic acid, which not only acts as a reducing agent but also stabilizes the highly energetic sharp protruding spikes of GNS (second step of Scheme 1).

### Synthesis of GNS Having High Aspect-Ratio Sharp Spikes

The traditional surfactant-free GNS synthesis was first developed by our group, which involves the simultaneous addition of AgNO_3_ and ascorbic acid to a solution of HAuCl_4_, HCl, and gold seeds at the concentration for GNS evolution.^29^ The morphology of surfactant-free GNS was tuned by systematically changing the reagent concentrations, including HAuCl_4_, ascorbic acid, AgNO_3_, HCl, and gold seeds. Recently, we have modified our GNS synthesis protocol by increasing the time gap to 5 s between the addition of AgNO_3_ and ascorbic acid, which would improve the reproducibility and homogeneity in the size distribution of the surfactant-free GNS.^36^ In the present work, HARS-2 and HARS-5 were synthesized. HARS-2 was synthesized by following our reported procedure, whereas the HARS-5 synthesis is a modified version of the original protocol by systematically controlling the concentration of the reagents (HAuCl_4_, HCl, ascorbic acid, and AgNO_3_) to achieve the highest number of spikes and sharp branches. Interestingly, we have seen that the reproducibility and branch sharpness was improved when the growth solution containing HAuCl_4_, HCl, and seeds was aged for 2 min, followed by the addition of AgNO_3_ and ascorbic acid with around a 5 s time gap. We believe that the aging of the growth solution for 2 min might help to get close contact with Cl^–^ ions, which facilitate the fine-tuning of the branches.

**Figure 1:**
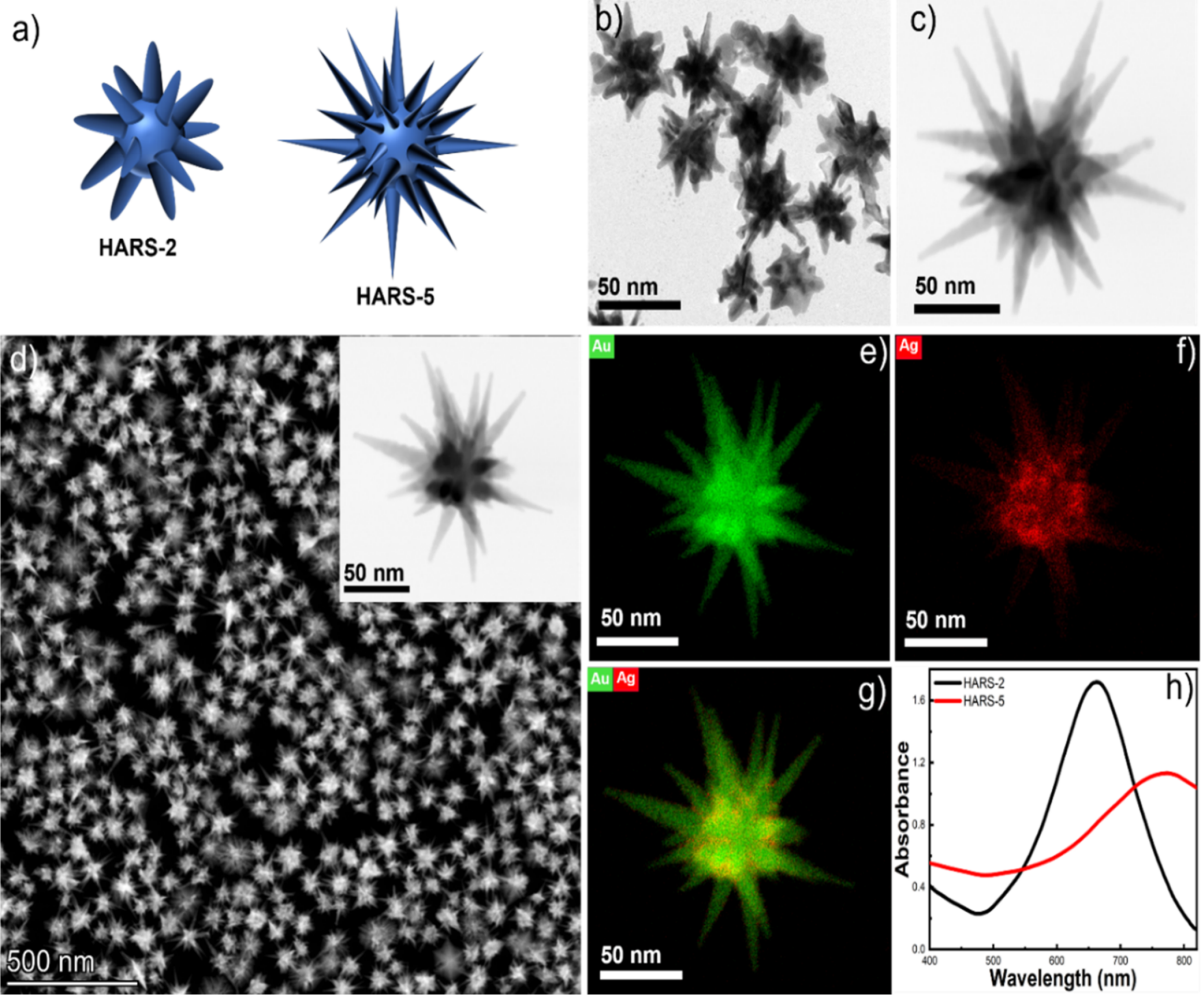
3D representation of HARS-2 and HARS-5 GNS (a). TEM image of HARS-2 (b) and STEM (bright field) image of HARS-5 (c). STEM image having multiple nanostars, including a STEM (bright field) image of HARS-5 (inset), which exhibits that the GNS synthesis is highly monodispersed (d). EDS elemental mapping of HARS-5 with sharp tips nanostructure showing silver is evenly distributed on the GNS (e–g). UV–vis spectra of GNS showing that the plasmon resonance maximum is redshifted from 680 nm (for HARS-2) to 780 nm (HARS-5) (h).

**Figure 2:**
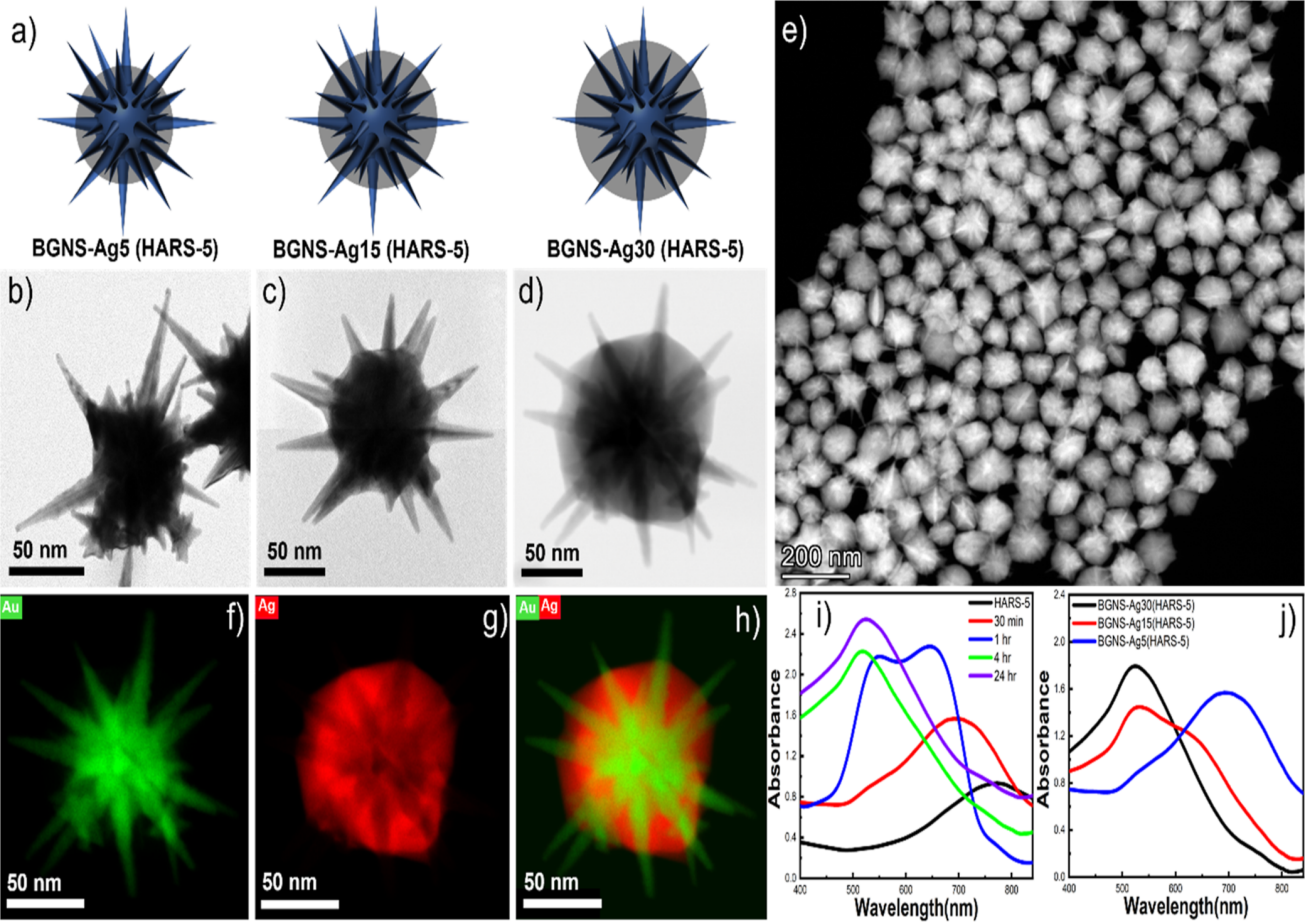
3D model of BGNS-Ag5, BGNS-Ag15, and BGNS-Ag30 for HARS-5 morphology (a) and their corresponding TEM image of BGNS-Ag5 and BGNS-Ag15 (b,c) and STEM (bright field) image of BGNS-Ag30 (d). STEM image having multiple nanostars (e). EDS elemental mapping of BGNS-Ag30, showing a thick silver layer is deposited on the core of GNS (f–h). UV–vis spectra (normalized) of BGNS-Ag, which exhibits that the GNS plasmon peak is blueshifted with time after the addition of 0.75 mM of AgNO_3_ (i). UV–vis spectra (normalized) of different thicknesses of silver on HARS-5 (j).

Figure 1a displays the 3D models of HARS-2 and HARS-5, and their corresponding TEM image for HARS-2 and STEM (bright field) image of HARS-5 was shown in Figure 2b,c, respectively, which reveals that the HARS-5 has the highest number of spikes and spike sharpness than HARS-2. The detailed morphological analysis of HARS-2 and HARS-5, including their core size, spike length, overall (tip-to-tip) size, spike aspect ratio, and spike sharpness, is summarized in Table 1. To analyze the distribution of elements of the HARS-5, energy dispersive X-ray spectroscopy (EDS) elemental mapping has been carried out. Figure 1e–g displays the EDS mapping where the green color indicates Au and the red color indicates Ag. It is found that the Ag is evenly distributed on the surface of the HARS, which is evident that a thin layer of Ag is deposited on the nanostars and stabilizes the complex GNS morphology.^40^Figure 1h displays the LSPRs of HARS-2 and HARS-5, which shows that the highest plasmon resonance peak of HARS-2 and HARS-5 was 680 and 780 nm, respectively. The optical property of the GNS is strongly dependent on the aspect ratio of HARS due to their LSPR. Therefore, the higher the aspect ratio, the plasmon resonance will get more redshifted.

**Table 1:** Morphological Characterization of HARS-2 and HARS-5

	number of spikes	tip to tip distance (nm)	core size (nm)	spike length (nm)	spike aspect ratio	Spike sharpness	λ_max_ (nm)
HARS-2	7–8	110 ± 15	70 ± 20	18 ± 8	1.8	7 ± 2	680
HARS-5	20–35	135 ± 20	47 ± 15	53 ± 12	4.7	3 ± 2	780

### Synthesis of BGNS-Ag

It is reported that an alkaline medium is necessary for the silver coating of GNS where the GNS was stabilized by surfactant CTAB.^37,38^ However, an alkaline medium would cause aggregation of surfactant-free GNS as the high aspect-ratio sharp branch morphology is highly delicate toward any change in the reaction medium, such as the pH and temperature of the medium.^41−43^ Therefore, it is important to develop an improved method for the silver coating of surfactant-free GNS to retain the morphology of highly delicate sharp spikes. Interestingly, it is reported that ascorbic acid can also be utilized as a reducing agent to reduce silver on gold nanoparticles without introducing alkaline NaOH or ammonia solution.^44^ In this study, we have investigated the silver coating of HARS morphology by using ascorbic acid as a reducing agent. However, we observed that there was no silver coating on HARS-5 even though we select a very high concentration of ascorbic acid (10 mM) and a high ratio of ascorbic acid and AgNO_3_ (6:1). We believe as the pH of the HARS-5 solution is very low (pH - 2.1), the reduction efficiency of ascorbic acid is not enough to reduce silver and deposit on the nanostars. To solve this issue, we diluted the nanostar solution with Milli-Q so that the pH of the solution was increased from 2.1 to 2.6, and ascorbic acid can slowly reduce AgNO_3_. The maximum silver coating on HARS-5 was achieved when the concentration of ascorbic acid and AgNO_3_ was 5 and 0.75 mM, respectively. Whereas for the HARS-2 morphology, the concentration of ascorbic acid and AgNO_3_ were 1–0.15 mM, respectively.

The characteristics of the resulting silver-coated HARS-2 [BGNS-Ag14 (HARS-2)] particles are shown in Figure S1. Figure S1a,b depicts the 3D model and TEM image of silver-coated HARS-2 [BGNS-Ag (HARS-2)], which reveals that the core size was increased from 90 to 104 nm, indicating silver deposition on the core, which was further confirmed by EDS analysis. The LSPR band of HARS-2 appears at 680 nm, and it is blueshifted to 500 nm after silver coating (Figure S1c), which is in good agreement with previously reported results.^37^ The silver coating was confirmed by STEM–EDS analysis, which is displayed in Figure S1d–g where the green color indicates Au, and the red color indicates Ag. The EDS images show that the Ag is preferentially deposited on the core of HARS-2, and the average thickness of silver is 14 nm.

For the HARS-5 morphology, we have synthesized three different BGNS morphologies by changing the concentration of AgNO_3_ from 0.3 to 1.5 mM. The 3D models (Figure 2a) and their corresponding TEM images (Figure 2b,c) and STEM (bright field) of the BGNS morphology (Figure 2d) show that the core size was increased 5 nm for BGNS-Ag5, 15 nm for BGNS-Ag15, and 30 nm for BGNS-Ag30 of HARS-5 (Figure 2d). Figure 2e displays the high monodispersity of the synthesis of BGNS-Ag30. The EDS images (Figure 2f–h) show that the Ag is preferentially deposited on the core of HARS-5, and the average thickness of silver is 30 nm. The silver coating was accompanied by the blueshifting of the LSPR. Figure 2i illustrates that the LSPR of BGNS-Ag30 was gradually blueshifted from 780 to 525 nm after 24 h. Interestingly, the blueshift shows a quite linear dependence on the Ag shell thickness where the main LSPR band of BGNS-Ag15 and BGNS-Ag-5 was 531 and 700 nm, respectively (Figure 2j). Figure S2 shows the high batch-to-batch reproducibility of the HARS-5 and BGNS-Ag 30 (HARS-5). However, the exact mechanism for the preferential deposition of silver on the core of GNS and detailed morphological analysis of these BGNSs will be further investigated in future studies.

### SERS Study and Reproducibility of SERS Performance

**Scheme 2:**
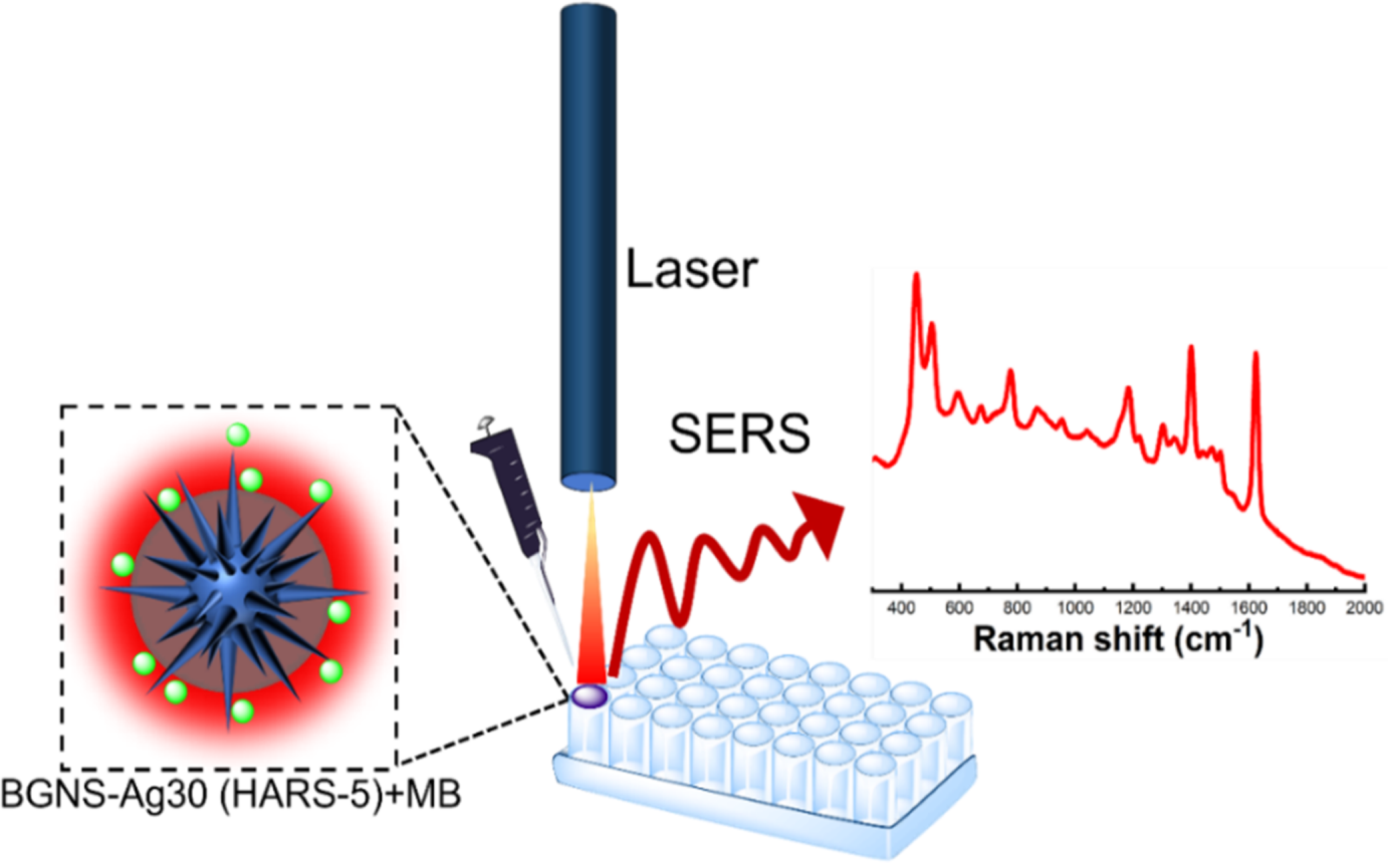
Schematic Representation of the Solution-Based SERS Detection Method of MB

Our SERS detection strategy for the solution-based SERS detection with minimal sample preparation is illustrated in Scheme 2, where we streamlined our SERS detection method by just mixing the SERS probe with the as-synthesized GNS in 96 well plates and performed the SERS measurement by using a handheld Raman instrument with a 785 nm laser to excite the analyte molecule.

**Figure 3:**
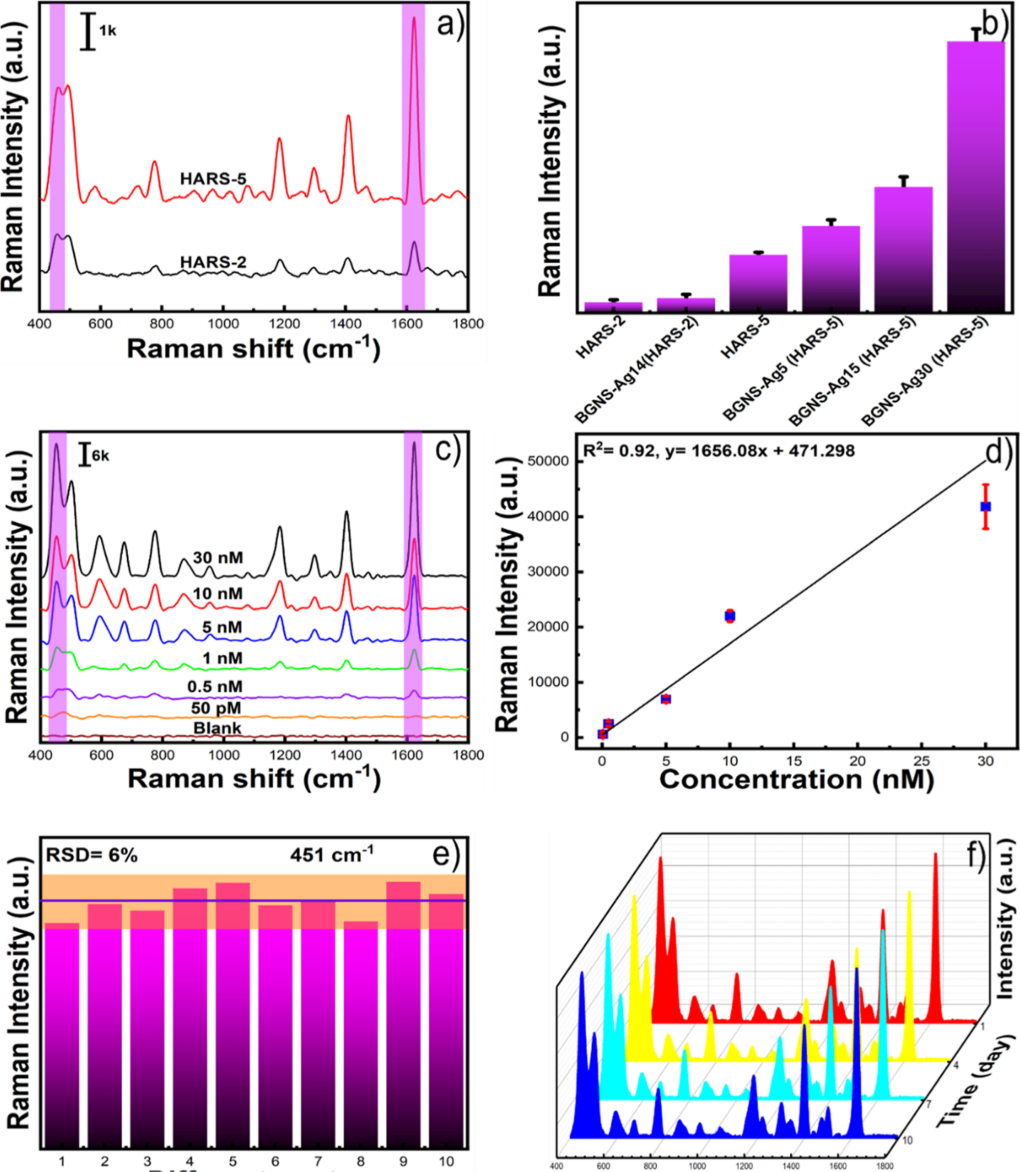
SERS spectra of MB (10 μM) for HARS-2 and HARS-5 (a). Comparison of the SERS signal intensity of MB at 451 cm^–1^ for HARS-2, BGNS-Ag-14 (HARS-2), BGNS-Ag5 (HARS-5), BGNS-Ag15 (HARS-5), and BGNS-Ag30 (HARS-5) (b). SERS spectra of MB for BGNS-Ag30 (HARS-5) with the concentration ranging from 30 nM to 50 pM (c). SERS peak intensity of MB at 451 cm^–1^ from 30 nM to 50 pM concentrations (d). SERS reproducibility of BGNS-Ag30 (HARS-5) (e). SERS stability of BGNS-Ag30 (HARS-5) (f).

In this work, the SERS performance of GNS was first investigated using methylene blue (MB) dye as a probe molecule at 10 nM concentration. Figure 3a shows the SERS signal intensity for HARS-5 at 451, 504, 774, 1193, 1298, 1394, and 1623 cm^–1^ of MB is far larger than that of HARS-2. This can be attributed to the fact that HARS-5 morphology has many sharp spikes and a higher surface area available for the binding of analyte molecules. As a result, multiple hot spots generate for HARS-5, which enhances the SERS performance.

Furthermore, we have investigated the SERS performance of silver-coated HARS-2 and HARS-5 morphology, where 10 measurements were carried out for a 10 nM concentration of MB (Figure 3b). Interestingly, we have observed that the intensity of the Raman peak of MB at 451 cm^–1^ was enhanced only for a 1.2 factor of silver-coated HARS-2, whereas BGNS-Ag30 shows five times stronger SERS enhancement than HARS-5. It is noteworthy that the SERS enhancement of MB was increased almost linearly with increasing the silver shell thickness of BGNS. As anticipated, BGNS-Ag30 (HARS-5) shows the highest SERS enhancement than others because it has high aspect-ratio sharp spikes, as well as a thicker silver layer than BGNS-Ag15 (HARS-5) and BGNS-Ag5 (HARS-5).

We have chosen BGNS-Ag30 (HARS-5) to investigate further the sensitivity of MB and another dye molecule, rhodamine 6G (R6G), and a thiolated small organic molecule 4-mercaptobenzoic acid (MBA). As shown in Figures 3c, S3, and S4, we have calculated the limit of detection (LOD) for MB, R6G, and MBA up to 42 pM, 45 pM, and 0.15 nM. Figure 3c is a plot of the intensity of the 451 cm^–1^ bands versus concentration. We have performed five Raman measurements for each concentration of MB. The calibration curve exhibited a linear relationship between the intensity and different concentrations of MB. Figures S3 and S4 showed that a linear relationship was observed also for the other two analytes (R6G and MBA). In addition, we have calculated the enhancement factor (EF) value of the BGNS-Ag30 (HARS-5)
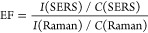
where *I*(SERS) and *I*(Raman) represent the intensity of SERS spectra and normal Raman spectra and *C*(SERS), and *C*(Raman) represents the concentration of analytes, respectively. We have selected the peak intensity at 451 cm^–1^ for MB, 1512 cm^–1^ for R6G, and 1592 cm^–1^ for MBA to determine the EF. Figure S5 shows the Raman spectra of MB, R6G, and MBA. The *C*(SERS) for the concentration of MB, R6G, and MBA are 50 pM, 50 pM, and 10 nM, respectively. The *C*(Raman) for the concentration of MB, R6G, and MBA are 0.5, 0.5, and 100 mM, respectively. The EF for BGNS-Ag30 (HARS-5) was calculated to be 1.1 × 10^6^ for MB, 5.6 × 10^6^ for R6G, and 1.2 × 10^7^ for MBA. These EF values are relatively high than the reported values.^20^ The high EF value of BGNS-Ag30 (HARS-5) is attributed to the factors both the HARS morphology and thick silver coating.

To verify the reproducibility, a SERS performance was carried out using an MB solution with a concentration of 10 nM as a probe molecule, and 10 different spots were chosen containing BGNS-Ag30 (HARS-5). Figure 3e displays the SERS signal heights at 451 cm^–1^. The relative standard deviation was calculated to be 6%, which indicates that the solution-based SERS detection method is highly reproducible. Moreover, we have investigated the stability of the substrate. Figure 3f shows that there is almost no difference in the SERS spectral profiles, which indicates that the BGNS-Ag30 (HARS-5) is stable over 10 days. In conclusion, we have achieved ultra-high sensitivity and good reproducibility of solution-based SERS measurement.

### Applications of BGNS-Ag for Pesticide Thiram Analysis

**Figure 4:**
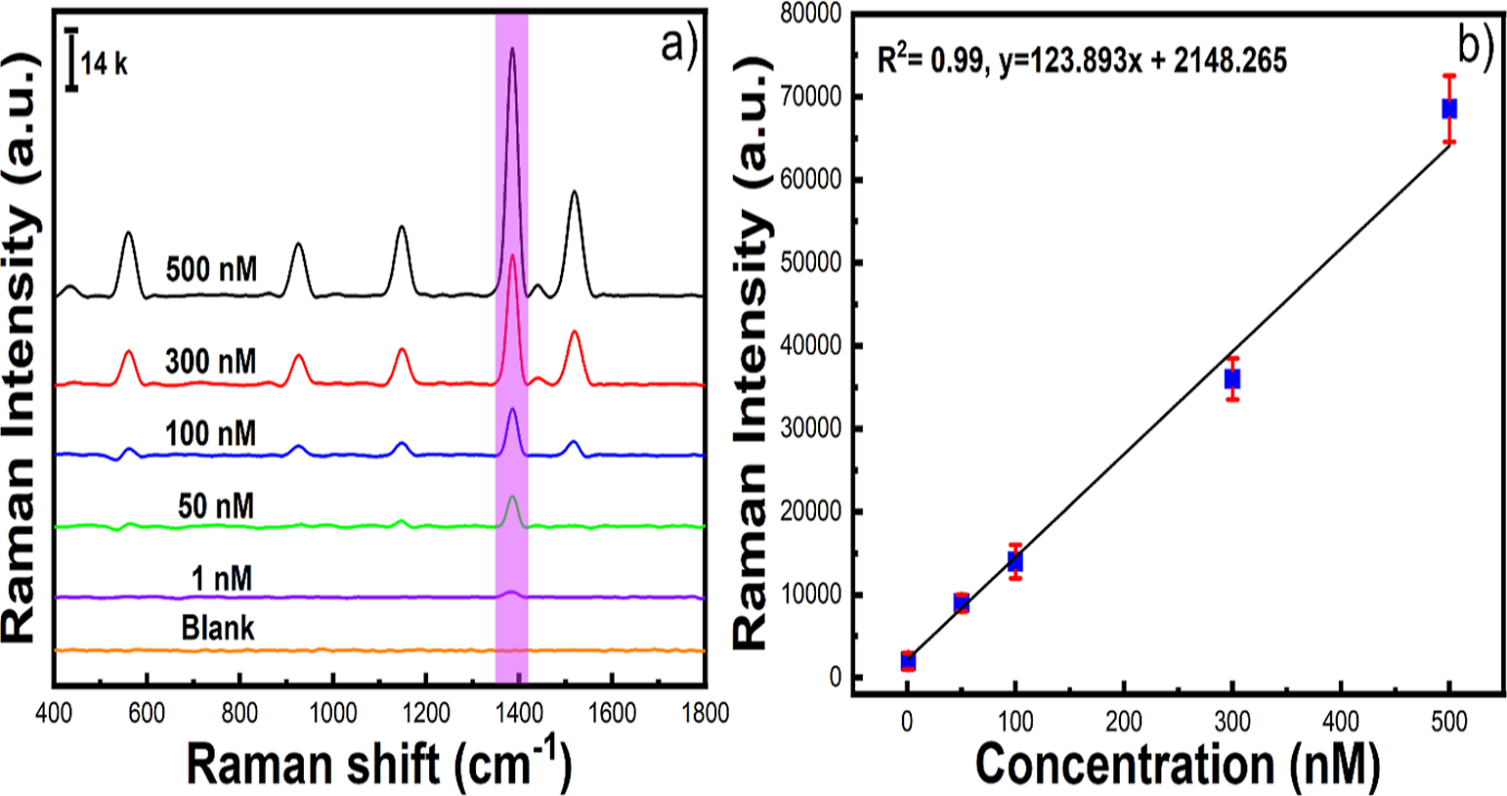
Solution-based SERS detection of thiram using BGNS-Ag30 (HARS-5) with concentrations ranging from 1 μM to 1 nM (a) and the peak intensity at 1381 cm^–1^ as a function of different concentrations (b).

We have investigated our solution-based SERS substrate method by using BGNS-Ag30 for detecting thiram, which is a toxic chemical and widely used as a pesticide for plant pest control. Figure 4 shows the SERS spectra of thiram at 1 μM concentration exhibiting the characteristic peaks at 561, 925, 1147, 1381, and 1507 cm^–1^, which are in agreement with reported values.^45^ Using the main characteristic band at 1381 cm^–1^ in the SERS spectrum, thiram can be detected to be as low as 0.8 nM. The extremely low LOD of thiram by using BGNS-Ag30 (HARS-5) and the use of a portable Raman instrument underlines the advantage of our proposed method over other solution-based or solid substrate-based SERS detection methods previously reported in the literature.^18,46,47^

## Conclusions

In summary, we have shown that surfactant-free GNS having a high aspect ratio and densely branched spikes can function as an efficient plasmonic template, and its silver coating can provide the unique morphology of the gold nanostructures to achieve ultrahigh SERS detection sensitivity by using a portable Raman instrument. The synthesis of BGNS-Ag is easy and offers an efficient and low-cost process for highly sensitive SERS substrates with good reproducibility. We have used MB, R6G, and MBA as probe molecules for our study and achieved detection limits as low as 42 pM, 45 pM, and 0.15 nM for MB, R6G, and MBA, respectively. To illustrate the usefulness of the SERS substrate for environmental sensing, we used the BGNS-Ag30 for detecting thiram, which is used as pest control for plants. The results showed that the LOD for thiram was 0.8 nM. The demonstrated advantages of the combination of SERS enhancements by silver coating on high aspect ratio sharp branched GNS opens new opportunities for simple, reliable, fast, and ultrasensitive SERS detection.

## Supporting Information Available

Experimental procedure, characterization of BGNS-Ag14 (HARS-2), UV–vis absorbance spectra of BGNS-Ag30 (HARS-5), SERS spectra of R6G and MBA using BGNS-Ag30 (HARS-5), and Raman spectra of MB, R6G, and MBA (PDF)
