# Supplementary material for: Bimetallic Gold Nanostars Having High Aspect Ratio Spikes for Sensitive Surface-Enhanced Raman Scattering Sensing
Source: ACS Appl Nano Mater. 2022 Aug 29;5(9):12562–70. doi: 10.1021/acsanm.2c02234 (PMC9513749; doi:10.1021/acsanm.2c02234)
Supplement: Supplementary file 1 — an2c02234_si_001.pdf [file an2c02234_si_001.pdf]

Supporting Information

Bimetallic Gold Nanostars Having High Aspect-Ratio Spikes for Sensitive Surface-Enhanced Raman Scattering Sensing

Supriya Atta,[a,b] Tuan Vo-Dinh[a,b,c] *

[a] Fitzpatrick Institute for Photonics, [b]Department of Biomedical Engineering, [c]Department of Chemistry, Duke University, Durham, NC 27708, US

**Corresponding Author: tuan.vodinh@duke.edu*

EXPERIMENTAL SECTION

Materials and Characterization

Chloroauric acid ($HAuCl_4$), L-ascorbic acid, silver nitrate ($AgNO_3$, 99.8%) hydrochloric acid (HCl), and trisodium citrate ($Na_3C_6H_5O_7$), methylene blue (MB), and rhodamine 6G (R6G), 4-mercaptobenzoic acid (MBA), and thiram were purchased from Sigma-Aldrich. Milli-Q deionized (DI) water was used throughout the experiment. The morphology of nanostars was characterized by analysis FEI Tecnai G2 Twin transmission electron microscope, and HAADF STEM images and EDS maps were acquired using Aberration Corrected STEM-Thermo Fisher Titan 80-300. UV-vis spectra were recorded using a Shimadzu UV-3600i spectrometer with cuvettes of 1 cm path length at room temperature.

Synthesis of HARS-2 Gold Nanostars

We have first synthesized 12 nm citrate capped gold seeds which was synthesized by following our previously reported method.[1] HARS-2 gold nanostars were synthesized by following our previously reported method.[1, 2] In this synthesis, we first added 50 μL of 1 N HCl to a solution of 50 mL of 0.25 mM $HAuCl_4$ and 500 μL of the citrate capped gold seed and the solution was stirred for 2 minutes. After that, we have added a solution of 250 μL of 3 mM $AgNO_3$ and 250 μL of 100 mM ascorbic acid to the growth solution with 5 second time interval. We have stirred the solution for 2 minutes which was used further for silver coating process.

Synthesis of HARS-5 Gold Nanostars

HARS-5 gold nanostars were synthesized by following our previously reported method.[1, 2] To synthesize HARS-5, we first added 200 μL of 1 N HCl to a solution containing 50 mL of 1 mM $HAuCl_4$ and 500 μL of Au seed solution which was further stirred for 2 minutes. After that, we have added a solution of 2mL of 3 mM $AgNO_3$ and 1 mL of 100 mM ascorbic acid to the solution with 5 second time interval and the solution was stirred for 2 minutes which was used further for silver coating process.

Synthesis of Bimetallic Silver Coated Gold Nanostars (BGNS-Ag).

The as-synthesized surfactant-free GNS solution was first diluted with 40 mL Milli-Q. Then, for HARS-5, 5 mM ascorbic acid and 0.75 mM $AgNO_3$ were added to the nanostars solution and stirred for 24 hours. Whereas for HARS-2 morphology, the concentration of 1 mM ascorbic acid and 0.15 mM $AgNO_3$ were added to the nanostars solution stirred for 24 hours.

For BGNS-Ag5 (HARS-5), the concentration of ascorbic acid and $AgNO_3$ were 1 mM and 0.15 mM respectively. For BGNS-Ag15 (HARS-5), the concentration of ascorbic acid and $AgNO_3$ were 3 mM and 0.45 mM respectively.

Raman Measurements

The Raman measurement was performed by following our previously reported method where we have used a Laser power of the Rigaku Xantus TM-1 at 150 mW and exposure time was set at 1 s.[2] For sample preparation, we have added 300 μL of gold nanostars with 3μL of analyte solution in 96 well plate. A small piece of aluminum foil was placed at the bottom of the 96 well plate to prevent signal interference of the polymer polypropylene well plate.

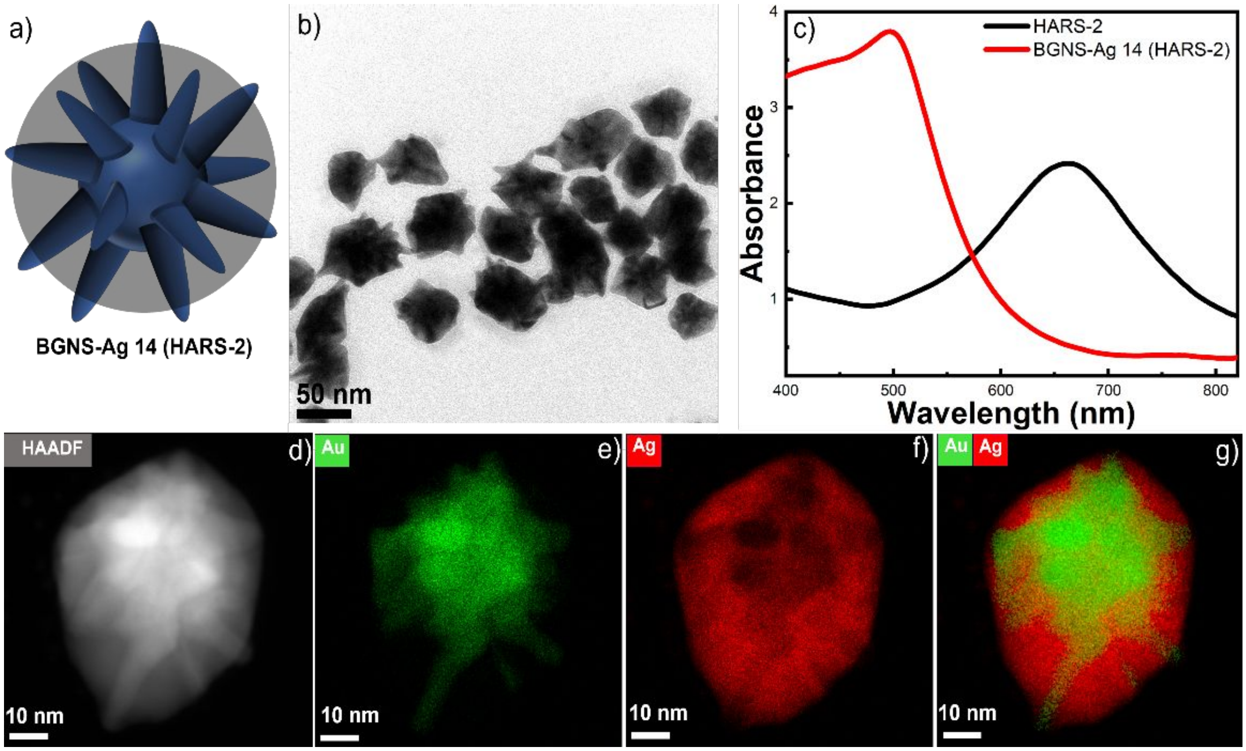

Figure S1. The 3D model and TEM image of BGNS-Ag14 (HARS-2) (a-b). UV-vis spectra showing a gradual blue shift of the HARS-2 plasmon peak from 680 nm to 500 nm after silver coating (c). EDS elemental mapping of BGNS-Ag14 (HARS-2) (d-g).

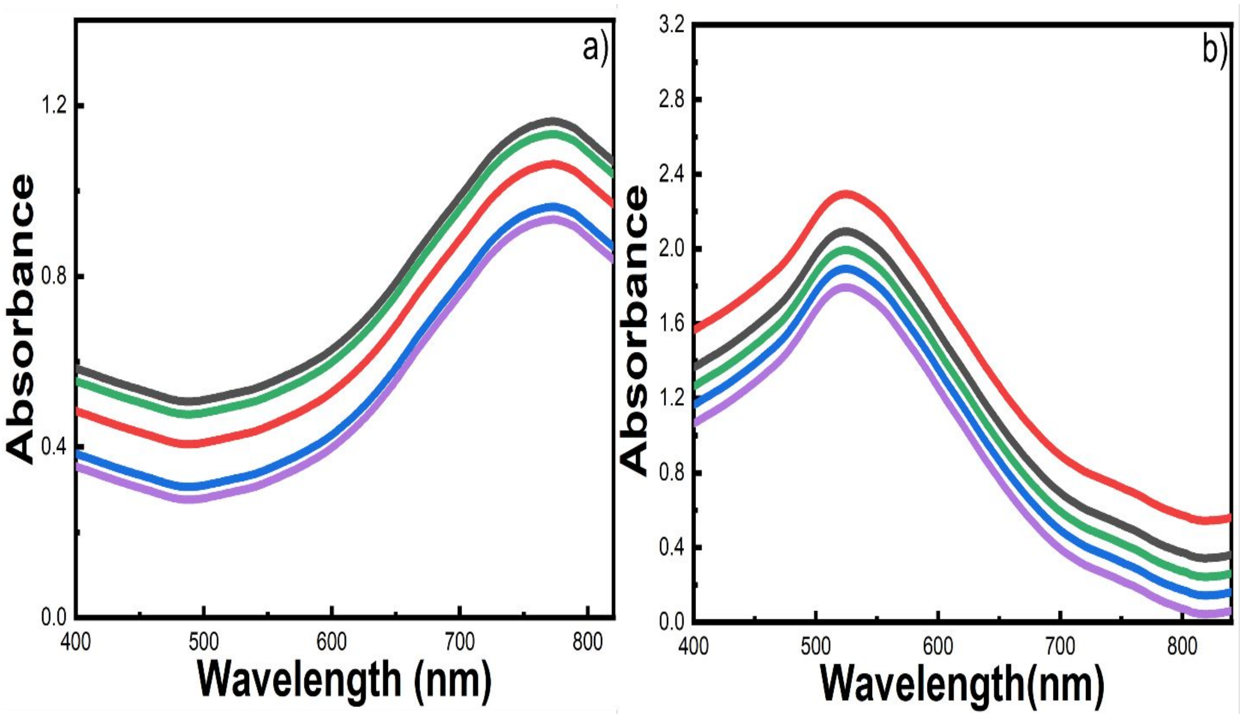

Figure S2. UV-Vis absorbance spectra of five different batches of HARS-5 (a) and BGNS-Ag30 (HARS-5) (b) which shows that these nanostars are highly reproducible.

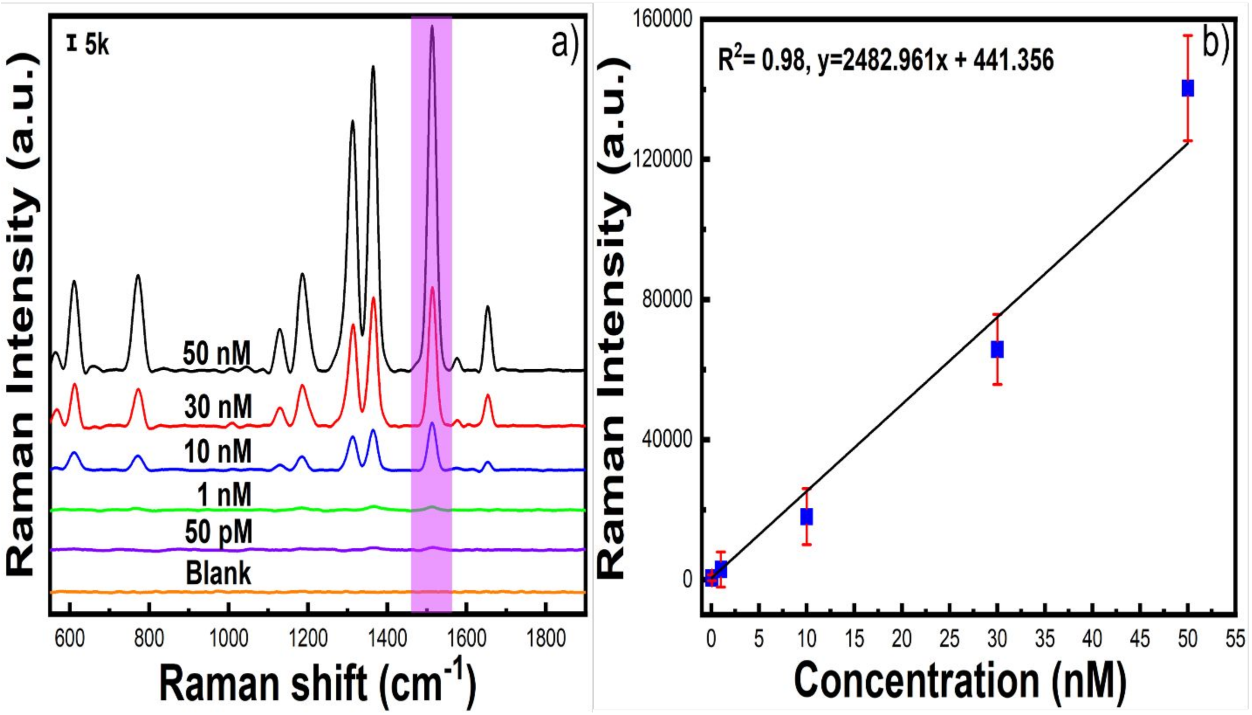

Figure S3. Solution-based SERS detection of R6G using BGNS-Ag30 (HARS-5) with

concentrations ranging from 50 nM to 50 pM (a), and the peak intensity at 1512 cm^{-1} as a function of different concentrations (b).

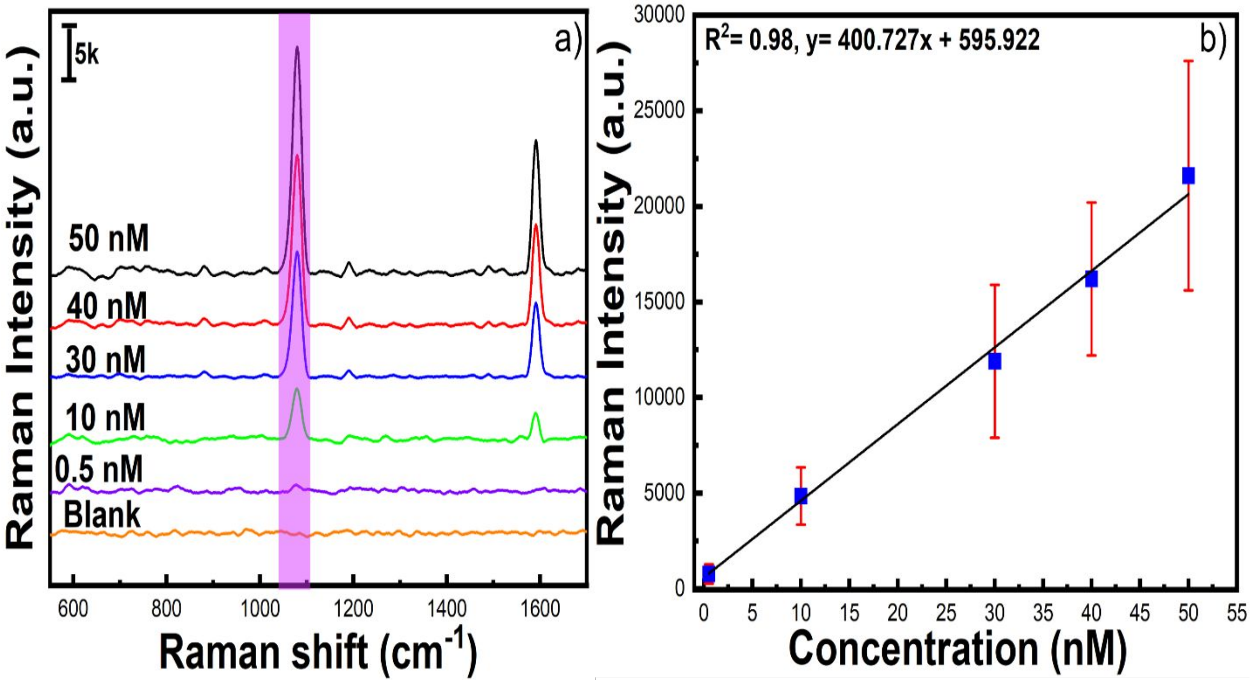

Figure S4. Solution-based SERS detection of MBA using BGNS-Ag30 (HARS-5) with concentrations ranging from 50 nM to 0.5 nM (a), and the peak intensity at 1078 cm^{-1} as a function of different concentrations (b).

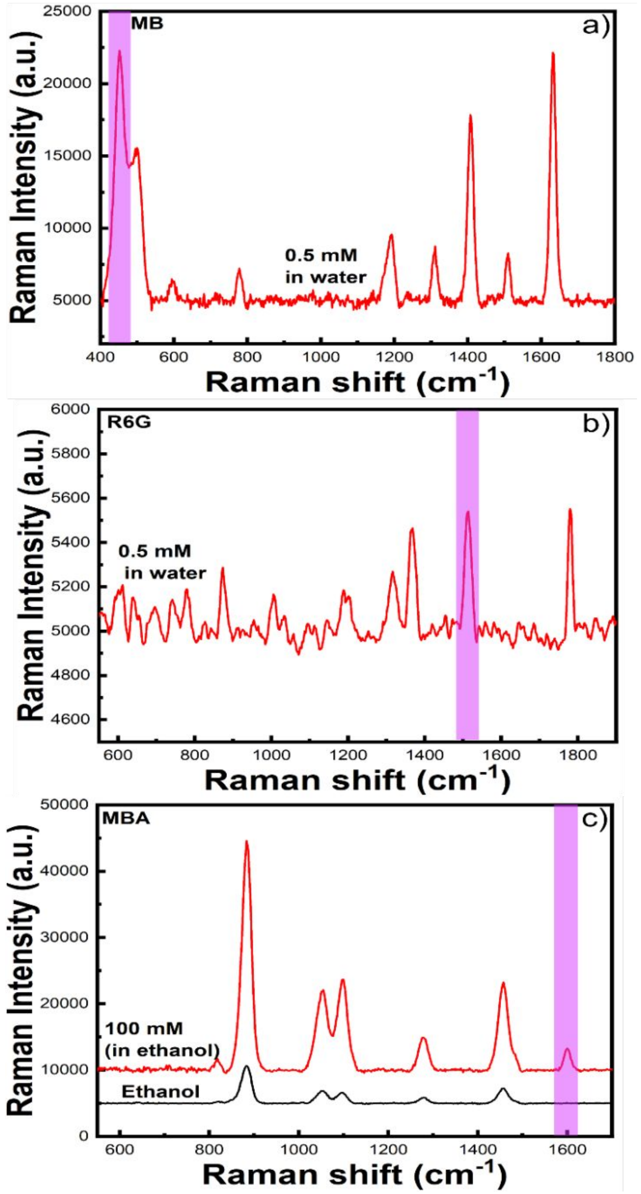

Figure S5. Raman spectra of MB at 0.5 mM concentration (a). Raman spectra of R6G at 0.5 mM concentration (b). Raman spectra of MBA at 100 mM concentration (c).

References.

1. Yuan, H.; Khoury, C. G.; Hwang, H.; Wilson, C. M.; Grant, G. A.; Vo-Dinh, T., Gold nanostars: surfactant-free synthesis, 3D modelling, and two-photon photoluminescence imaging. *Nanotechnology* **2012,** *23* (7), 075102.
2. Atta, S.; Watcharawittayakul, T.; Vo-Dinh, T., Ultra-high SERS detection of consumable coloring agents using plasmonic gold nanostars with high aspect-ratio spikes. *Analyst* **2022,** *147* (14), 3340-3349.
